# Subclinical varicose vein recurrence: modern approaches in aesthetic phlebology

**DOI:** 10.1590/1677-5449.202501622

**Published:** 2026-03-23

**Authors:** Serhii Shchukin

**Affiliations:** 1 Bogomolets National Medical University, Kyiv, Ukraine.

**Keywords:** varicose veins, recurrence, telangiectasis, sclerotherapy, laser therapy, ultrasonography, Doppler, endovascular procedures, augmented reality, CLaCS (cryo-laser and cryo-sclerotherapy), varizes, recorrência, telangiectasia, escleroterapia, terapia a laser, ultrassonografia doppler, procedimentos endovasculares, realidade aumentada, CLaCS (criolaser e crioescleroterapia)

## Abstract

**Background:**

Recurrence after varicose vein treatment is usually framed as varices on a previously treated limb, potentially overlooking patients with cosmetic C0–C1 concerns who still experience duplex Doppler ultrasound-verified reflux.

**Objectives:**

To characterize patterns of subclinical (C1-dominant) recurrences in duplex Doppler ultrasound and evaluate a 2-stage pathway prioritizing hemodynamic correction before aesthetic care.

**Methods:**

This retrospective single-center cohort study (2018-2024) was conducted with patients from a vascular clinic. The participants were 354 women with unaesthetic telangiectasias/reticular veins without C2 varices on the index limb after prior varicose treatment. The interventions (exposures) were: (Stage 1) ultrasound-guided 3% polidocanol foam for segments ≤ 3 mm and endovenous laser ablation ≥ 3 mm (anterior accessory saphenous vein/ greater saphenous vein /small saphenous vein/perforators); (Stage 2) 2 sessions of cryo-laser and cryo-sclerotherapy at a 1-month interval, guided by near-infrared augmented-reality. The main outcomes were duplex-verified occlusion of Stage-1 targets at 1 month and aesthetic outcome at 6 months (rated on a 5-point Likert scale). Safety measures included symptomatic venous thromboembolism and minor adverse effects.

**Results:**

At 1 month, all targets were occluded and no symptomatic venous thromboembolisms had occurred. Mapping frequently showed multilevel reflux (anterior accessory saphenous vein, angiomatosis in the vein stump or strip tract, junctional complexes, perforators), with 78% of patients having ≥ 2 sources. At 6 months, good/excellent aesthetic outcomes (Likert scale score 4-5) were achieved in 83.1% of patients (mean score: 4.22). The scoring distribution was: 1 point: 7 (2.0%); 2 points: 11 (3.1%); 3 points: 42 (11.9%); 4 points: 131 (37.0%); and 5 points: 163 (46.0%). Minor events were limited to transient pigmentation/matting.

**Conclusions:**

Subclinical recurrence is chiefly hemodynamic. An etiology-first strategy using duplex Doppler ultrasound followed by cryo-laser and cryo-sclerotherapy yields high early satisfaction and could mitigate early cosmetic recurrence.

## Resumo

**Contexto:**

Recorrência pós-tratamento de varizes é geralmente classificada como C2 em membro previamente tratado, potencialmente negligenciando pacientes com queixas estéticas C0–C1 que ainda apresentam refluxo verificado por dúplex.

**Objetivos:**

Caracterizar padrões dúplex de recorrências subclínicas (C1-dominante) e avaliar uma abordagem de duas etapas, priorizando correção hemodinâmica pré-tratamento estético.

**Métodos:**

Coorte retrospectiva unicêntrica em clínica vascular ambulatorial (2018–2024). Participantes: 354 mulheres com telangiectasias/veias reticulares inestéticas sem varizes C2 no membro inicialmente tratado pós-tratamento venoso prévio. Etapa 1: espuma de polidocanol 3% guiada por ultrassom para segmentos ≤3 mm; EVLA para ≥3 mm (VSAA/VSM/VSP/perfurantes). Etapa 2: CLaCS (criolaser + crioescleroterapia) em duas sessões com intervalo de um mês, guiado por realidade aumentada em infravermelho próximo. Principais desfechos e medidas. Primários: oclusão por dúplex dos alvos da Etapa 1 em 1 mês e desfecho estético em 6 meses (Likert, 5 pontos). Segurança: TEV sintomático e efeitos adversos menores.

**Resultados:**

Em 1 mês, todas as veias-alvo estavam ocluídas; nenhum TEV sintomático ocorreu. Mapeamento mostrou frequentemente refluxo multinível (VSAA, angiomatose de coto/faixa, complexos juncionais, perfurantes); 78% tinham ≥2 fontes. Em 6 meses, resultados bons/excelentes (4–5) em 83,1% (média 4,22). A distribuição das pontuações Likert foi: 1 ponto: 7 (2,0%); 2 pontos: 11 (3,1%); 3 pontos: 42 (11,9%); 4 pontos: 131 (37,0%); 5 pontos: 163 (46,0%). Eventos menores foram limitados à pigmentação/*matting* transitória.

**Conclusões:**

Recorrência subclínica é predominantemente hemodinâmica. Estratégia guiada por dúplex, priorizando etiologia, seguida por CLaCS, produz alta satisfação precoce e pode mitigar a recidiva estética precoce.

## INTRODUCTION

Modern minimally invasive methods for treating varicose vein disease have progressively replaced traditional traumatic surgeries (ligation of the saphenofemoral junction, stripping, phlebectomy). In most phlebology clinics worldwide, thermal and non-thermal ablation techniques and ambulatory phlebectomy are routine procedures. Nevertheless, the problem of recurrence remains pertinent: according to systematic reviews and randomized studies, the recurrence rate after various methods ranges from 7%–32%.^1-4^ At a consensus meeting on this topic, recurrence was clinically defined as the presence of varicose veins on a lower limb that has previously undergone surgery for varicose veins.^5^ Based on this definition, one might conclude that no varicose veins equals no recurrence. However, a significant number of female patients complain about the appearance of unsightly veins on their legs after varicose vein removal. In duplex Doppler ultrasound (DUS) scanning, new refluxes are frequently detected in such patients, feeding spider veins (telangiectasias and reticular veins). It is appropriate to consider such cases as subclinical recurrences — ultrasonographically verified sources of pathological reflux. Telangiectasias and reticular veins are a fairly common phenomenon in the legs. The prevalence of telangiectasias and reticular veins in the general population is high, ranging from 60% to 86%; they can be isolated or associated with varicose veins (C2 according to clinical, etiologic, anatomic, and pathophysiologic classification).^6^

For this category of patients, cosmetics is the primary concern, but the success of aesthetic treatment directly depends on the correct identification and elimination of underlying feeding veins/refluxing segments, particularly perforators and accessory saphenous trunks. Otherwise, recurrent lesions or post-sclerotic matting quickly develop.^7^ This approach correlates with current guidelines, which emphasize the necessity of DUS for every recurrence and before aesthetic interventions to determine the sources of reflux.^3^

For patients with predominantly C1 class veins (telangiectasias and reticular veins), a recent Cochrane review indicated that no single method (injection sclerotherapy or laser) was clearly more effective, and data on recurrences in this group remain limited. Combined approaches have shown better aesthetic outcomes but require further validation.^8^

In contrast, for hemodynamically relevant refluxing segments, endovenous thermal ablation has demonstrated durable anatomical and clinical results. Long-term prospective data using 1470-nm radial fiber EVLA have confirmed stable occlusion of the great and small saphenous veins, supporting its role as a cornerstone of etiological treatment in recurrent disease.^9^

In the aesthetic treatment of C1-class veins, cryo-laser and cryo-sclerotherapy (CLaCS) has emerged as a combined technique. A recent randomized controlled trial demonstrated superior clearance of telangiectasias and reticular veins with CLaCS compared with conventional sclerotherapy, with significantly less pigmentation.^7^ In addition, the cryolaser-after-foam technique has been reported as a feasible and effective option in clinical practice.^10^ Augmented-reality vein visualization (VeinViewer) has been introduced to improve identification and targeting of subcutaneous feeding veins during CLaCS procedures.^11^

Despite the large number of studies on recurrence after treatment for trunk vein incompetence, subclinical (C1-dominant) recurrence is described only sporadically. Underestimating this etiological component increases the risk of rapid recurrent lesions, pigmentation, or matting after monotherapy for superficial C1 class veins.^11^

## OBJECTIVE

This purpose of this study was to develop an algorithm for the staged management of patients with subclinical recurrence of varicose vein disease and to evaluate the results of combined treatment of C1-class varicose vein recurrence using minimally invasive techniques.

## MATERIALS AND METHODS

Design and Population. This retrospective single-center cohort study included 354 patients with subclinical (C1) recurrences after previous surgical treatment for varicose vein disease at our medical center between 2018 and 2024. All patients were female, aged 22–61 (mean 42.16 [SD, 12.35]) years; the time since primary treatment was 4.2 (SD, 1.8) years. The primary complaint was spider veins (telangiectasias and reticular veins) in the absence of clinically significant C2 disease.^1,2^

### Inclusion criteria

Patients who underwent surgical intervention for varicose vein disease, spider veins in the lower extremities that are cosmetically undesirable, and in whom DUS revealed venous reflux (feeding veins).

**Figure 1:**
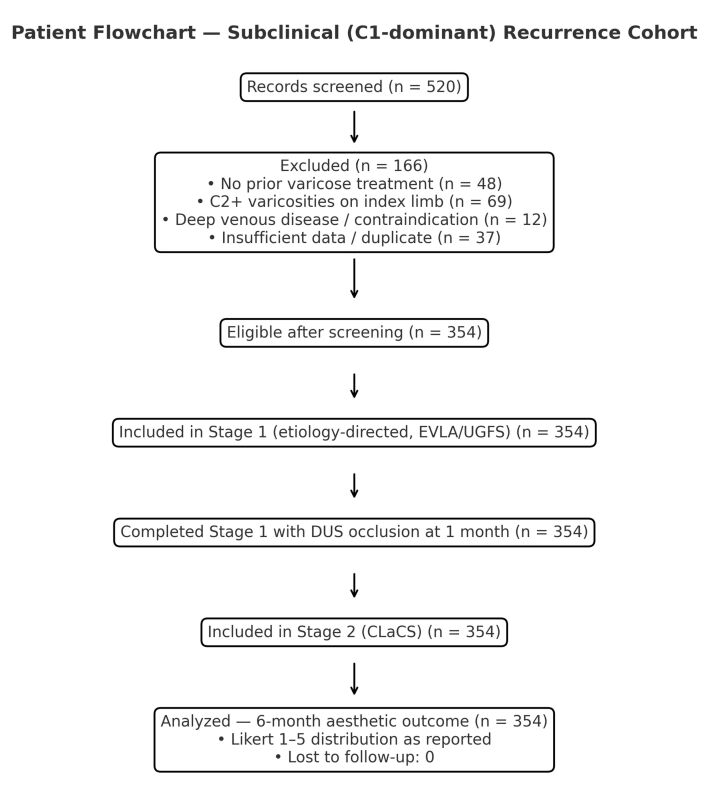
Flow diagram of patient selection and inclusion in the study cohort.

*Exclusion criteria*. Patients whose lower-limb varicose veins had been surgically treated (traditional "clinical" recurrences) were not included in this cohort. The inclusion and exclusion process is illustrated in Figure 1.

### Definitions

Subclinical recurrence was considered the reappearance or persistence of a source of venous reflux (detected by DUS) after previous treatment, with no clinically significant varicose nodes and predominantly C1-class disease (telangiectasias and reticular veins).

### Duplex doppler ultrasound protocol and provocative maneuvers

DUS was performed with the patient standing and shifting her body weight to the contralateral leg; the examined limb was in slight external rotation, with the knee relaxed. Room temperature was maintained at approx. 22 °C–24 °C to prevent vasospasm. Ultrasound examination was performed using an Esaote MyLab Seven device with a 7 MHz–15 MHz linear transducer. Spectral Doppler settings included: angle ≤ 60 °, sample volume size 2 mm, low-frequency filter, and a sufficient sweep speed for accurate measurement of reflux duration.

Provocative maneuvers included Valsalva (or brief cough) at the saphenofemoral junction, distal manual compression release for superficial trunks/tributaries, active tip-toe/calf-pump at the saphenopopliteal junction/small saphenous vein, targeted perforator testing with proximal or distal compression (or a 60–80 mmHg short cuff), and proximal thigh compression (or Valsalva) for deep veins. Reflux thresholds were > 0.5 s at superficial junctions/segments, > 0.35–0.5 s for outward perforator flow, and > 1.0 s for the deep system. All maneuvers were performed using minimal probe pressure and with the patient standing. Full DUS mapping of feeding veins was performed, assessing the saphenofemoral and saphenopopliteal junctions, accessory anterior saphenous vein, tributaries, and perforators, as well as signs of lymph node varicosities in the vein stump or strip tract. Multiple sources were identified in 276 (78%) patients.

### Stage 1 – reflux correction

Areas of angiomatosis in the anterior accessory saphenous vein, great saphenous vein, small saphenous vein, and perforating veins with a diameter < 3 mm underwent echo-sclerotherapy. For sclerotherapy, 1%-3% polidocanol foam (1 mL–3 mL per injection) was used. The Tessari technique or the double-syringe system was employed to obtain stable, finely dispersed foam. This involved mixing 1 mL of polidocanol with 2-3 mL of air, agitated 20–30 times using a two-syringe system connected by a 3-way stopcock.

EVLA was performed if the diameter of the anterior accessory saphenous vein, greater saphenous vein, small saphenous vein, or popliteal vein was > 3 mm. EVLA was performed using a Biolitec Ceralas diode laser device (Biolitec, Vienna, Austria) with an Elves radial 2-ring fiber, at 6 W-7 W, with automated fiber retraction at 0.7 mm/s. The linear endovenous energy density was maintained at 70 J/cm-90 J/cm. For tumescent anesthesia, a chilled buffered 0.1% lidocaine solution without adrenaline was used.

### Stage 2 – aesthetic correction

CLaCS for reticular veins and telangiectasias was performed twice for each limb 1 month and 2 months after Stage 1 treatment had been completed. To mitigate selection and measurement bias, we used a standardized DUS protocol with predefined reflux thresholds and verified Stage-1 occlusion prior to CLaCS. For cutaneous laser treatment, an Nd:YAG 1064 nm Fotona XP Dynamis laser system (Fotona, Ljubljana, Slovenia) with cryo-cooling (Zimmer Cryo 6; Zimmer MedizinSystem, Neu-Ulm, Bavaria, Germany) and near-infrared vein visualization (VeinViewer Vision 2; Christie Medical Holdings, Inc., Lake Mary, FL, USA) was used for precise targeting of feeding veins.^7,11,12^ Telangiectasias were visualized using a 4x headlamp/loupe system with polarized light illumination (Syris 900v; Syris Scientific, Gray, ME, USA). Skin cooling was performed using a Zimmer Cryo 6 cryocooler. Additional contact cooling was applied.

### First CLaCS Session

Reticular vein treatment protocol: cutaneous laser treatment included spot size 9 mm, fluence –50 J/cm^2^, pulse duration 35 ms.

Sclerotherapy: injections of 0.3% polidocanol (Aethoxysklerol; Kreussler Pharma, Wiesbaden, Germany). The 3% polidocanol solution was diluted 10-fold with 40% glucose. The liquid form was used for sclerotherapy.

Telangiectasia treatment protocol: cutaneous laser treatment included spot size 4 mm, fluence – 160 J/cm^2^, pulse duration 5 ms.

After each procedure, Class 2 compression stockings were prescribed for 1 day.

Second CLaCS session (1 month after the first session)

Reticular vein treatment protocol: cutaneous laser treatment included spot size 6 mm, fluence – 75 J/cm^2^, pulse duration 15 ms.

Sclerotherapy was performed as in the first session.

### Telangiectasia treatment protocol

Cutaneous laser treatment: spot size 2 mm, fluence – 360 J/cm^2^, pulse duration 0.6 ms, with 3-4 passes performed. It is essential to use the Syris 900v headlamp/loupe system for timely detection of purpura onset along the telangiectasia and prevent burns.

After each procedure, Class 2 compression stockings were prescribed for 1 day.

### Post-procedural care and follow-up

Control DUS 1 was performed month after Stage 1 to confirm occlusion of the target segments.

### Endpoints

Primary:

1. DUS-verified occlusion of target segments at 1 month;
2. Aesthetic outcome at 6 months, assessed using a 5-point Likert scale.

Secondary: pigmentation/matting, need for additional sessions, adverse events.

### Statistical methods

We analyzed a consecutive convenience sample; no a priori sample-size calculation was performed. Internal consistency checks were performed; duplicates were removed prior to analysis.

Sample-size adequacy (single proportion). Based on Miot’s framework for precision and assuming a 6-month good/excellent rate of p≈0.83, a two-sided 95% CI with ± 4%–5% precision would require n≈338–216. Our cohort (n=354) exceeded these thresholds, ensuring ≤ 4% precision (95% CI).^12^

Data distributions were assessed visually and with the Shapiro-Wilk test. Continuous variables were summarized as mean (SD) (for approximately normal distributions) or as median [IQR] (for nonnormal distributions). Categorical variables are presented as n (%).

Five-point Likert scale ratings were treated as ordinal. We present the full distribution of categories and the median (IQR); the mean is provided only as a descriptive characteristic. For intergroup comparisons, Mann-Whitney U (2 groups) or Kruskal-Wallis with post-hoc Dunn-Bonferroni (≥ 3 groups) was applied. The Wilcoxon signed-rank test was used for paired data.

Proportions were compared using the χ^2^ test (with Yates' correction if needed) or Fisher's exact test if expected the frequencies were < 5. The 95% CI for proportions were calculated using Wilson's method. All tests were two-sided, with α = 0.05. Missing data were not imputed (complete-case analysis).

Sources of reflux were described in a per-limb/lesion-level format: since one limb could have several sources, the proportions by category do not sum to 100%. Percentages were calculated with the denominator as the number of limbs studied (n = 354). The number of procedures in Stages 1 and 2 was reported as the number of interventions performed, not the number of patients. No subgroup or sensitivity analyses were planned.

### Ethical considerations

This study, conducted in accordance with Declaration of Helsinki guidelines and current national regulatory requirements, was approved by the Bogomolets National Medical University Bioethics Committee (Protocol 186; Jun. 24, 2024). It was conducted and reported in accordance with Strengthening the Reporting of Observational Studies in Epidemiology (STROBE) recommendations.^13^ The completed STROBE checklist is provided as Supplementary Material.

### Study Registration

Given that this was a retrospective observational study, registration was not applicable, although it was subject to reporting guidelines and oversight. The manuscript adheres to STROBE recommendations (checklist and flowchart submitted).

### Study results

#### Duplex doppler ultrasound confirmation after stage 1

At 1 month, all target segments were occluded according to DUS results; no thromboembolic complications were recorded. Minor side effects were limited to transient hyperpigmentation and occasional matting in the aesthetic correction zone; no skin necrosis, nerve damage, or infections were observed.

#### Duplex doppler ultrasound mapping patterns in subclinical (C1-Dominant) recurrences

Systematic mapping revealed a predominance of multilevel feeding pathways instead of a single "culprit." The most frequent combinations included the anterior accessory saphenous vein, often along with varicose angiomatosis of the greater saphenous vein stump), junctional neovascular complexes, and incompetent perforators (Thierry/Hach). Reflux was observed in infrapopliteal pathways along the small saphenous vein trunk or its femoral "extension," with contributions from calf perforators. In 78% of patients, mapping indicated ≥ 2 concurrent sources, which explains why isolated aesthetic correction without an etiological investigation often results in recurrence; see **Table 1.**

**Table 1:** Sources of reflux, based on the etiology first, cosmetics second strategy*.

**Source of reflux**	**Prevalence, n (% of limbs, *n*=354)**	**Stage 1: etiology‑directed correction (US‑guided)**	**Stage 2: aesthetic correction**
Inguinal lymph node angiomatosis	**78 (22.1%)**	EVLA of refluxing trunk/tributary (often the AASV) ≥ 3 mm; targeted US‑guided 3% polidocanol foam into the angiomatous complex	CLaCS + liquid 0.5% polidocanol for residual RV/TAE
GSV stump angiomatosis	**92 (26.0%)**	Targeted 3% foam into stump neovascularization; EVLA of residual GSV segment if present	CLaCS over the network
Strip‑tract angiomatosis	**78 (22.1%)**	Chain injections of 3% foam along the tract; EVLA of direct reflux segments where present	CLaCS on residual C1 elements
AASV	**106 (29.9%)**	**EVLA n=75;** EVLA of AASV (≥ 3 mm) from the confluence; add 3% foam to tributaries if needed	CLaCS of reticular branches/TAE
Thierry perforator	**64 (18.1%)**	**EVLA n=46;** EVLA of the perforator or 3% foam (per diameter/depth)	Local CLaCS
Preserved GSV segment on the thigh	**50 (14.1%)**	**EVLA n=36;** EVLA of the remnant GSV	CLaCS as needed
Hach perforator	**57 (16.1%)**	**EVLA n=41;** EVLA of the perforator or 3% foam	Local CLaCS
SPJ angiomatosis with ascending reflux via femoral extension of SSV	**43 (12.2%)**	EVLA of proximal SSV and femoral extension; adjunct 3% foam into the angiomatosis	CLaCS on calf/thigh
Pudendal reflux	**28 (7.9%)**	Targeted sclerotherapy of the pudendal plexus under VeinViewer/augmented reality	CLaCS in the perineal TAE area (if present)
SSV trunk reflux	**71 (20.1%)**	**EVLA n=50;** EVLA of the SSV (≥ 3 mm); adjunct 3% foam if needed	CLaCS on residuals

* Because the counts are lesion-level (ie, a single limb could contribute multiple sources), the totals in each row are non-additive. AASV: anterior accessory saphenous vein; augmented reality: augmented reality; CLaCS: Cryo Laser Cryo Sclerotherapy; EVLA: Endovenous Laser Ablation; GSV: greater saphenous vein; RV: reticular vein; SPJ: saphenopopliteal junction; SSV: small saphenous vein; TAE: telangiectasias; US: ultrasound.

### Aesthetic outcomes after stage 2

**Figure 2:**
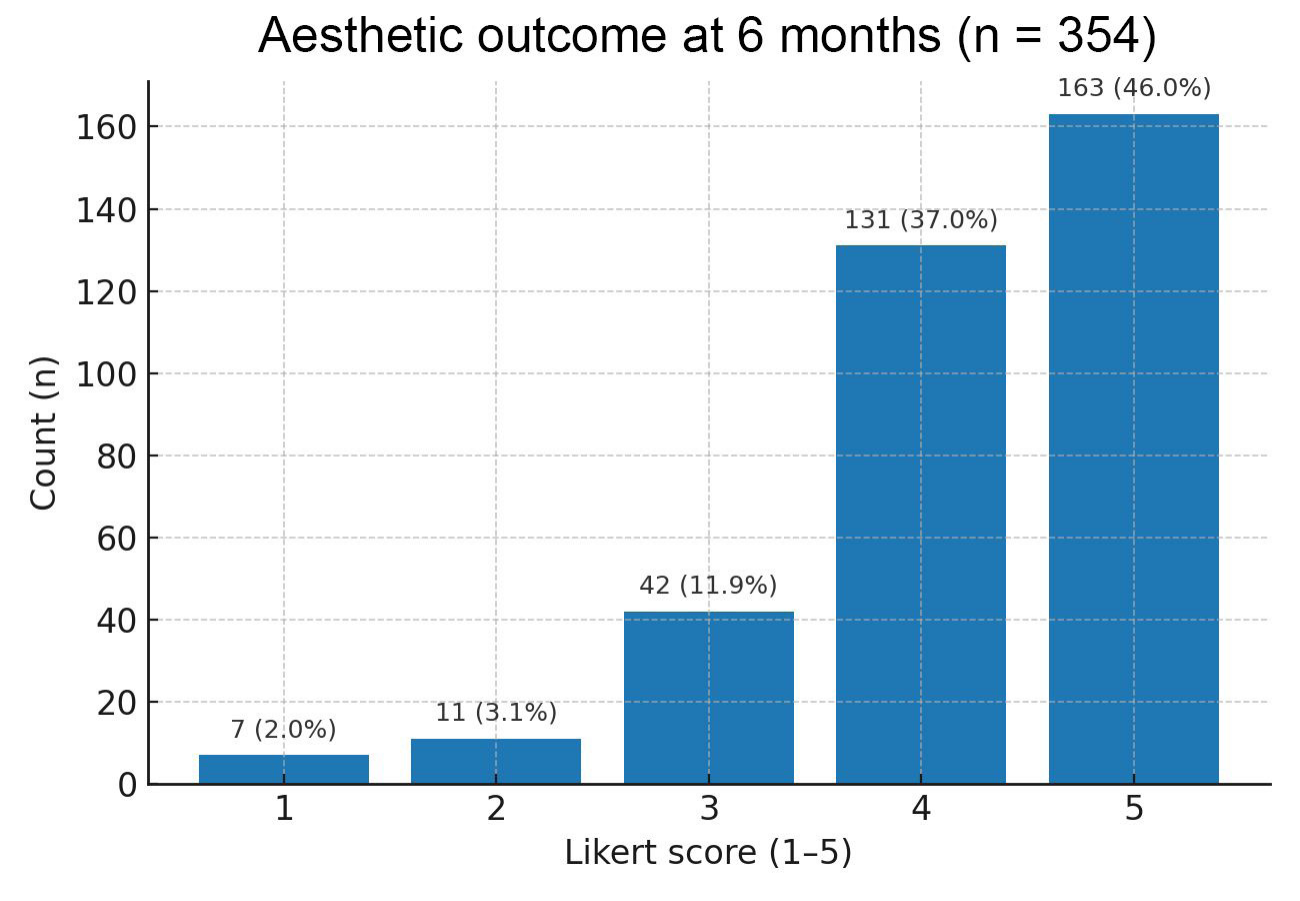
Distribution of aesthetic outcomes at 6 months (Likert scale scores; n = 354) with 95% Wilson confidence intervals.

CLaCS was performed twice per limb, with a 1-month interval between sessions. Six months after treatment completion, a 5-point Likert scale (n = 354) was applied, showing good/excellent (4–5 points) results in 294/354 (83.1%) of the patients (95% CI: 78.8–86.6; mean score: 4.22)(see Figure 2).

### Clinical interpretation

Patients scoring 2–3 points (n = 53; 15.0%) typically had a pronounced reticular network and required 3–5 sessions; those who scored 1 point (n = 7; 2.0%) had DUS-verified refluxes due to partial recanalization of areas that were sclerosed in the first stage. This outcome required additional ultrasound-guided sclerotherapy sessions before continuing aesthetic procedures. No recanalization was observed after EVLA.

## DISCUSSION

The traditional definition of recurrence effectively restricts it to C2 varices in a previously treated limb.^5^ Conversely, our data delineate an important category of ultrasound-detected (subclinical) recurrence, in which a source of reflux exists, but C1 disease predominates.

**Figure 3:**
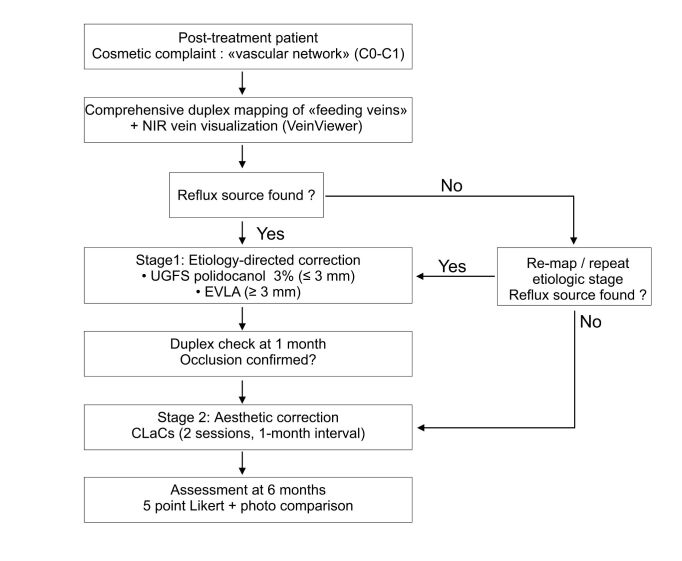
Management algorithm for subclinical (aesthetic) recurrences: etiology first, cosmetics second.

Our proposed two-stage algorithm (first correct the reflux, then address the superficial network) has demonstrated reproducible efficacy: the targeted elimination of reflux (EVLA/echo-sclerotherapy) ensures complete occlusion of target segments, while subsequent CLaCS provides sustained improvement in aesthetic outcomes with a low incidence of adverse events. Comprehensive DUS mapping is the key to identifying feeding veins (anterior accessory saphenous vein, perforators, angiomatosis of the vein stump or strip tract, pudendal reflux, etc.) and their targeted correction before the aesthetic stage.^14^Figure 3.

### Current concepts of recurrence after endovenous therapy

In the context of modern phlebology, the evolution of varicose vein treatment from traditional surgical interventions to minimally invasive endovenous methods has significantly improved outcomes and reduced invasiveness. However, the issue of recurrence remains relevant.^15,16^ Contemporary research has identified a number of key factors contributing to recurrence after endovenous ablation. Recurrence after endovenous therapy is mainly driven by residual or recanalized segments, persistent/overlooked anterior accessory saphenous vein incompetence, and post-procedural junctional neovascularization. Additional contributors include progression in untreated below-knee segments^17^ and incompetent perforator veins, which re-establish superficial reflux.

Specifically, Bacchelieri et al. emphasize the significance of both anatomical peculiarities and organizational aspects in recurrence after radiofrequency ablation.^4^ This highlights the critical importance of precise preoperative mapping and standardization of procedures to minimize errors that can lead to recurrence.

Cong et al. demonstrated that hybrid laser strategies effectively reduce the recurrence frequency in below-knee vein segments compared to radiofrequency ablation monotherapy in real-world clinical practice.^17^ This underscores the particular vulnerability of distal venous segments and the need for an individualized approach to adapting energy parameters to the anatomical features of the knee and lower leg.

Our approach, based on the priority use of DUS for planning and controlling interventions, is supported by two key technical justifications:

1. First, the application of flush thermal ablation combined with sclerotherapy of saphenofemoral junction tributaries demonstrates enhanced control over the proximal zone in recurrent cases.^18^ This strategy effectively eliminates residual feeding veins, which otherwise could manifest as telangiectasias or reticular veins (C1 networks). This approach minimizes residual reflux at the junction level and its closest tributaries.
2. Second, clinical feasibility studies have confirmed that EVLA remains an effective and appropriate method, even in cases of morphologically complex recurrences. This applies to recurrences in the saphenofemoral junction area, as well as after previous operations on the small saphenous vein, provided careful individualized access planning and adaptation of energy parameters according to detailed preoperative ultrasound mapping. This indicates the high adaptability of EVLA to the complex anatomical conditions of recurrence.^14-16^

### The role of sclerotherapy: focus on ultrasound guided foam sclerotherapy for trunks and tributaries

Sclerotherapy holds a significant place in the arsenal of modern varicose vein treatment methods, especially in the context of small-diameter and tortuous venous segments. Among the various types of sclerotherapy, ultrasound guided foam sclerotherapy (UGFS) merits particular attention as a highly effective etiological tool for occluding affected veins.

The effectiveness of UGFS is supported by data from contemporary multicenter studies. Specifically, a low 5-year recurrence rate and significant improvement in patient quality of life after UGFS of vein trunks have been demonstrated. However, a key prerequisite for achieving such results is standardizing the technique and reaching a clear DUS endpoint—namely, non-compressive occlusion of the treated vein.^14^

These scientific data fully align with our clinical protocol, which stipulates a differentiated approach to selecting the ablation method based on vein diameter. According to this protocol:

- UGFS is applied in veins ≤ 3 mm in diameter or for areas of angiomatosis.
- EVLA is preferred for veins ≥ 3 mm diameter.

In any case, the key is to achieve a clear DUS endpoint after ablation, which serves as the criterion for procedural success. Only after this is achieved do we proceed to aesthetic aspects. This strategic approach ensures maximum efficacy in long-term treatment outcomes.

### Aesthetic stage: evidence for cryo-laser and cryo-sclerotherapy with augmented reality navigation

Aesthetic correction of visible telangiectasias and reticular veins is an integral part of comprehensive treatment for chronic venous diseases, aimed at improving quality of life. Modern approaches in this domain are evolving, using advanced technologies to optimize outcomes.

In a triple-blind randomized controlled trial (PG3T), Bertanha et al. compared 0.2% polidocanol + 70% glucose vs 75% glucose alone for treating purely aesthetic telangiectasias (C1 class), demonstrating superior clearance rates with the combined agent (82.2% vs 63.9%) and no major adverse events.^19^

However, their study focused exclusively on aesthetic microvascular work (reticular and telangiectatic veins) without eliminating sources of reflux. In contrast, our staged protocol combines a hemodynamic-first correction followed by CLaCS in the aesthetic phase. In CLaCS, the transdermal Nd:YAG laser acts externally through the adventitia, media, and endothelium, inducing controlled photothermal injury of all 3 vascular wall layers, which ensures homogeneous fibrosis and gradual vessel resorption. This mechanism, described histologically by Miyake (2014), enhances the sclerosing action of dextrose by causing edema of the tunica media and prolonging the agent’s contact with the vein lumen.^20^ As a result, low-concentration polidocanol can be safely used as an adjuvant, minimizing thrombus formation and preventing hemosiderin pigmentation and capillary hypertension (matting). Furthermore, 70%–75% of glucose formulations are not registered in Ukraine, making CLaCS both a technically and pharmaceutically optimal approach in our clinical context.

In the context of treating telangiectasias and reticular veins, randomized controlled trials have demonstrated the superiority of CLaCS over isolated sclerotherapy. This method allows for a more pronounced aesthetic effect due to the synergistic action of laser energy and a sclerosing agent, administered under local cooling.

Real-world clinical practice has also confirmed the advisability of using the cryo-laser-after-foam technique for resistant venous networks.^10^ This approach has proven feasible and safe, providing an effective solution for complex cases that do not respond to standard methods.

Furthermore, augmented reality technologies are becoming increasingly important for accurate visualization and targeted treatment of subcutaneous feeding veins. Experience from individual clinical cases indicates significant improvement in the precise management of these vessels, leading to stable and long-lasting aesthetic results. This fully aligns with our practice of using augmented reality visualization before performing laser procedures, through which hidden veins can be visualized, ensuring their targeted ablation.^11^ Nevertheless, augmented reality does not replace DUS mapping, which remains the gold standard for verifying sources of reflux and stratifying tactics.^1,3^

### Interpretation of the results in a clinical context

Our patient cohort data indicate significant progress in treating recurrent forms of varicose disease. Specifically, we achieved complete DUS-verified occlusion of target feeding veins 1 month after the intervention, which is a key criterion for the technical success of the procedure. Furthermore, at 6 months of follow-up, 83% of the patients reported good or excellent aesthetic results.

Although our observation period was somewhat shorter than other series evaluating the effectiveness of thermal methods, our 2-stage treatment strategy fully aligns with current clinical recommendations.^3,7,9^ We hypothesize that such an approach can effectively reduce the risk of early cosmetic recurrence, particularly by minimizing the formation of matting and recurrent venous networks through the elimination of their etiological factors.

The predominance of combined therapy with EVLA and UGFS reflects the objective reality of DUS. In our patient group, 78% had multi-source reflux, which is rarely amenable to correction with a single therapeutic instrument. This fully correlates with reports from other researchers on the frequent co-existence of contributions from saphenofemoral junction tributaries, the anterior accessory saphenous vein, and popliteal vein in the pathogenesis of recurrence. Moreover, our results confirm data on the higher durability of thermal occlusion of large-diameter veins, which is a significant argument in favor of adding EVLA into the combined treatment regimen.^4,9,15,18^

### Practical implications and clinical recommendations

Considering our results and current data in the literature, we have formulated a series of key practical implications that should be integrated into clinical practice to optimize varicose vein disease treatment:

1. Mandatory DUS scanning prior to aesthetic interventions: before any aesthetic procedures aimed at correcting telangiectasias (spider veins), a detailed DUS scan is absolutely mandatory. The purpose of such an examination is a thorough search for hidden sources of reflux, since treating only visible manifestations without eliminating the underlying cause often leads to rapid recurrence.
2. A 2-stage approach prioritizing etiological treatment over cosmetic treatment: a sequence of therapeutic interventions that first involves eliminating the etiological factors of varicose disease (i.e., treating reflux in main trunks and perforators), and only then performing cosmetic correction, is fundamental. This approach significantly reduces the risk of early lesion recurrence and minimizes side effects such as pigmentation and matting (the formation of fine vascular networks).
3. Use of auxiliary visualization technologies: integrating modern auxiliary visualization technologies, such as near-infrared vein visualization, significantly enhances the targeting and accuracy of sclerotherapy. This allows for more effective identification and treatment of feeding veins that may be invisible to the naked eye. Consequently, the use of such technologies not only improves clinical results but also potentially reduces the overall required volume of sclerosing agent, minimizing risks and costs.

The strengths of this study include a unified treatment protocol, a homogeneous patient cohort (women with recurrences, predominantly class C1), and its reflection of real-world clinical practice. However, limitations remain, including its retrospective, single-center design, the subjectivity of the aesthetic assessment, the lack of a control group, and the lack of standardized objective scales. All of this underscores the urgent need for future prospective studies using validated instruments to evaluate cosmetic outcomes and long-term effectiveness.

## CONCLUSIONS

1. Preclinical recurrence, primarily manifesting as telangiectasias and reticular veins, is fundamentally a hemodynamic problem. Its occurrence is closely linked to persistent or newly formed pathological venous reflux, leading to increased venous pressure in the superficial venous system. Insufficient understanding of complex venous anatomy and hemodynamics, as well as tactical or technical errors during the primary intervention, can lead to the persistence of insufficiency and the formation of new reflux pathways.
2. Effective treatment of preclinical recurrence requires a DUS-guided, 2-stage approach, which involves sequential correction of:
  - pathological reflux in feeding veins using EVLA or UGFS;
  - cosmetic issues using the CLaCS method.
3. Such a sequential approach, focusing initially on eliminating etiological factors, ensures high early patient satisfaction and can reduce the frequency of early aesthetic recurrences.

## Data Availability

The data supporting the findings of this study are available upon request from the corresponding author, SS, due to ethical and privacy restrictions.

## Supplementary Material

Supplementary material accompanies this paper.

STROBE checklist (Cohort study) completed for a manuscript.

This material is available as part of the online article from https://doi.org/10.1590/1677-5449.202501622
