# Supplementary material for: Subclinical varicose vein recurrence: modern approaches in aesthetic phlebology
Source: J Vasc Bras. 2026 Mar 23;25:e20250162. doi: 10.1590/1677-5449.202501622 (PMC13020991; doi:10.1590/1677-5449.202501622)
Supplement: STROBE checklist (Cohort study) [file jvb-25-e20250162-suppl.pdf]

STROBE checklist (Cohort study) — completed for a manuscript

Study design: Retrospective single-center cohort (observational)

Manuscript title: **Subclinical Varicose Vein Recurrence: Modern Approaches in Aesthetic Phlebology**

Format: No. — Item (abridged requirement) | Where in manuscript | Status / Notes

1. Title & Abstract — Indicate study design; provide structured abstract.

| Title; Abstract | Done — "retrospective single-center cohort"; structured sections present.

2. Background/Rationale — Explain scientific background.

| Introduction (paras 1–3) | Done.

3. Objectives — State specific objectives/hypotheses.

| Introduction (last paragraph) | Done.

4. Study design — Present key elements early in Methods.

| Materials and Methods ? Study design and population | Done.

5. Setting — Describe setting, location(s), and dates.

| Study design and population | Done — ambulatory vascular practice; 2018–2024.

6. Participants — Eligibility criteria; sources and methods of selection.

| Inclusion and exclusion criteria | Done.

7. Variables — Define outcomes, exposures, predictors/confounders.

| Definitions; Endpoints; Duplex mapping and sources of reflux | Core done. Optional enhancement: list potential confounders (e.g., age, interval since index treatment) even if not modeled.

8. Data sources/measurement — Data sources; methods of assessment; comparability.

| Duplex scanning protocol and provocative maneuvers; Skin mapping | Done.

9. Bias — Describe efforts to address potential bias.

| Duplex protocol; Stage-1 verification before CLaCS | Partially covered. Add one sentence: "To mitigate selection/measurement bias, we used a standardized standing-duplex protocol with predefined reflux thresholds and verified Stage-1 occlusion before CLaCS."

10. Study size — Explain how study size was arrived at.

| Study design and population | Needs one sentence: "We analyzed a consecutive convenience sample; no a priori sample-size calculation was performed."

11. Quantitative variables — Explain handling/categorization.

| Definitions; Treatment strategy; Statistical methods | Done — UGFS ≤3 mm; EVLA ≥3 mm; Likert treated as ordinal.

12. Statistical methods — Describe all methods; handling of missing data; sensitivity analyses; subgroup analyses.

| Statistical methods | Done — Shapiro–Wilk; Mann–Whitney / Kruskal–Wallis with Dunn–Bonferroni; Wilcoxon; ??/Fisher; Wilson 95% CIs; two-sided ?=0.05; complete-case analysis. Add one sentence if desired: "No subgroup or sensitivity analyses were planned."

13. Participants (flow) — Report numbers at each stage; reasons for exclusions.

| Results ? Duplex confirmation; Flow diagram (submitted) | Done — n=354 included; primary endpoints available for all. Flow diagram file submitted.

14. Descriptive data — Give characteristics of participants.

| Study design and population (age; interval); Results ? Mapping patterns | Done. Optional: a small baseline table (age; interval since index treatment; side) strengthens this item.

15. Outcome data — Report numbers/summary measures for outcomes.

| Results ? Duplex confirmation; Aesthetic outcomes after Stage 2 | Done.

16. Main results — Provide estimates with precision (95% CIs); address confounding if applicable.

| Results ? Aesthetic outcomes; Figure 1 caption | Done/partial: full Likert distribution + 83.1%. Ensure Wilson 95% CI is explicitly stated either in text or figure caption (it is noted in your figure caption).

17. Other analyses — Subgroup/interaction; sensitivity.

| — | Not performed. Include explicit sentence in Methods or Discussion: "No subgroup or sensitivity analyses were planned."

18. Key results — Summarize key results with reference to objectives.

| Discussion ? opening paragraphs | Done.

19. Limitations — Discuss limitations, direction/magnitude of potential bias.

| Discussion ? Limitations / Interpretation | Done — retrospective, single center, women only, short follow-up, no control group.

20. Interpretation — Give cautious overall interpretation considering objectives, limitations, and related evidence.

| Discussion ? Overall interpretation; Practical implications | Done.

21. Generalisability — Discuss external validity of results.
| Discussion ? Practical implications / Generalisability | Done — applicability to aesthetic-dominant female ambulatory cohorts.
22. Funding — Source and role of funders.
| Funding; Conflicts of interest | Done — no specific funding; no conflicts of interest.

RECORD extension (Routinely Collected Data) — completed for your manuscript
R1. Data source — Indicate that data originated from routinely collected clinical/duplex records. Study design and population; Data availability Done.
R2. Data cleaning — Describe data cleaning methods.
| Study design and population / Statistical methods | Add one sentence: “Internal consistency checks were performed; duplicates were removed prior to analysis.”
R3. Codes/algorithms — Provide diagnostic/procedure codes or algorithms used to identify cases (if any).
Not applicable — case identification was chart/duplex-based without code filters.
R4. Linkage — Describe any database linkage (if performed).
Not applicable — no database linkage was performed.
R5. Access to protocol/analysis code — Where to access protocol or analysis plan/code. Data availability | Done/brief: “Protocol and analysis scripts available from the author upon reasonable request and approvals.”
